# Deep Learning Model Based on 3D Optical Coherence Tomography Images for the Automated Detection of Pathologic Myopia

**DOI:** 10.3390/diagnostics12030742

**Published:** 2022-03-18

**Authors:** So-Jin Park, Taehoon Ko, Chan-Kee Park, Yong-Chan Kim, In-Young Choi

**Affiliations:** 1 Department of Medical Informatics, College of Medicine, The Catholic University of Korea, Seoul 06591, Korea; sjpark2029@gmail.com (S.-J.P.); thko@catholic.ac.kr (T.K.); 2 Department of Biomedicine & Health Sciences, College of Medicine, The Catholic University of Korea, Seoul 06591, Korea; 3 Department of Ophthalmology, Seoul St. Mary’s Hospital, College of Medicine, The Catholic University of Korea, Seoul 06591, Korea; ckpark@catholic.ac.kr; 4 Department of Ophthalmology, Incheon St. Mary’s Hospital, College of Medicine, The Catholic University of Korea, Seoul 06591, Korea

**Keywords:** myopia, optical coherence tomography, deep learning, convolutional neural networks, transfer learning

## Abstract

Pathologic myopia causes vision impairment and blindness, and therefore, necessitates a prompt diagnosis. However, there is no standardized definition of pathologic myopia, and its interpretation by 3D optical coherence tomography images is subjective, requiring considerable time and money. Therefore, there is a need for a diagnostic tool that can automatically and quickly diagnose pathologic myopia in patients. This study aimed to develop an algorithm that uses 3D optical coherence tomography volumetric images (C-scan) to automatically diagnose patients with pathologic myopia. The study was conducted using 367 eyes of patients who underwent optical coherence tomography tests at the Ophthalmology Department of Incheon St. Mary’s Hospital and Seoul St. Mary’s Hospital from January 2012 to May 2020. To automatically diagnose pathologic myopia, a deep learning model was developed using 3D optical coherence tomography images. The model was developed using transfer learning based on four pre-trained convolutional neural networks (ResNet18, ResNext50, EfficientNetB0, EfficientNetB4). Grad-CAM was used to visualize features affecting the detection of pathologic myopia. The performance of each model was evaluated and compared based on accuracy, sensitivity, specificity, and area under the receiver operating characteristic curve (AUROC). The model based on EfficientNetB4 showed the best performance (95% accuracy, 93% sensitivity, 96% specificity, and 98% AUROC) in identifying pathologic myopia.

## 1. Introduction

The prevalence of pathologic myopia is 3% worldwide, and it is a leading cause of vision impairment in East Asian countries, such as China, Japan, Singapore, and Korea [1,2]. Pathologic myopia leads to complications and blindness, such as retinal detachment, cataracts, glaucoma, and macular degeneration [3,4]. Therefore, the timely diagnosis of pathologic myopia is crucial to prevent visual impairment and blindness in patients.

Pathologic and high myopia have somewhat confusing definitions, possibly due to the lack of a quantitative explanation of the differences between the two [5]. High myopia is defined as an anteroposterior elongation of the globe and a high level of myopia refraction error [6], while pathologic myopia is characterized by the occurrence of typical myopia lesions in the posterior fundus. According to the International Myopia Institute (IMI), the definition of pathologic myopia refers to a case where a structural change in the posterior eyeball globe occurs owing to the elongation of the axial length, resulting in abnormal eye function [6,7]. The term “myopia macular disease” has a similar concept to pathological myopia. In 1970, Curtin proposed definitions of myopia macular disease, including laryngeal atrophy, laryngeal spots, lacquer cracks, laryngeal staphyloma, and optic disc changes [8,9]. High myopia can be easily detected in most patients by measuring refractive errors that are routinely performed in ophthalmology, while many methods have been proposed to classify pathological myopia [10]. Since the introduction of fundus photography, a photographic classification system and optical consistency tomography criteria have been proposed. The qualitative analysis of pathological myopia using fundus photographs enables basic pattern recognition of diseases, but there is a limit to defining the degree of early diseases, which can be biased by subjective interpretation [11]. A qualitative analysis of pathological myopia using optical coherence tomography (OCT) is time-consuming and expensive because experts need to check a large number of three-dimensional images [12]. Most clinical doctors are specialists in a small group of diseases. Therefore, they have difficulties identifying diseases that are unfamiliar to their training background. If the corresponding disease is categorized in a subjective and ambiguous way, it makes the clinical doctor’s job more challenging. Pathologic myopia is a subset of ocular disease that is very poorly defined. Therefore, the development of an algorithm for the automatic diagnosis of pathologic myopia can be usefully applied as a clinical decision support system capable of identifying patients in a timely manner to help the clinicians that are unfamiliar with the disease.

Recently, deep learning technology using images has been developed in ophthalmology, and research on different diseases with various data (fundus photography, OCT, etc.) was conducted [13,14]. In particular, various deep learning techniques have been adopted to diagnose ophthalmic diseases using OCT images. Lin et al. used deep neural networks and random forest ensembles to understand the diagnostic performance of OCT B-scans. They compared the diagnostic performance of age-related macular degeneration, diabetic macular edema, and primary open-angle glaucoma with OCT images dividing the macular range [15]. Yoo et al. demonstrated that diagnosis accuracy improved using the generative adversarial networks for rare ophthalmic diseases [16]. Another study improved diagnostic accuracy and created a visually interpretable model through U-Net architecture and deep learning for classifying retinal diseases in 3D OCT images. This is also clinically applicable to images extracted from other devices [17]. Currently, there are studies that apply machine learning and AI technology to the diagnosis of pathologic myopia patients [10,11]. Pathologic myopia can be diagnosed using OCT and fundus images; however, OCT can confirm the 3D structure of the posterior and quantify the location of the disease occurrence, allowing for a more detailed and precise examination than fundus photography [11,18]. Pathologic myopia can be diagnosed using OCT [19], which requires a considerable amount of time and money from skilled experts. Therefore, there is a need for an algorithm that can clearly and simply diagnose pathologic myopia, by learning the posterior eyeball in a 3D structure without a segmentation process. 

This study is the first to develop an algorithm that automates the diagnosis of pathologic myopia using three-dimensional OCT images. The algorithm development uses 3D convolutional neural network (CNN)-based transfer learning, which is specialized in the field of image recognition and is suitable for learning spatio-temporal features. This will help to accurately identify patients with pathologic myopia, which can help reduce the risk of vision damage via the provision of timely treatment.

## 2. Materials and Methods

### 2.1. Study Population

This multicenter retrospective study was conducted using 1839 eyes of patients examined in the ophthalmology departments of Incheon St. Mary’s Hospital and Seoul St. Mary’s Hospital between January 2012 and May 2020. The conditions for exclusion of data used in the analysis were as follows: (1) Axl < 24.0 mm (*n* = 321), (2) other retinal or choroidal diseases, such as diabetic retinopathy, retinal vascular disease, or age-related macular degeneration (*n* = 45), (3) a history of vitreoretinal, glaucoma filtering, or tube surgery (*n* = 54), and (4) missing data or poor image quality (*n* = 1022). The total number of eyes included in the analysis was 367. Finally, the eyes were categorized into a normal group and a group with pathologic myopia, the number in each being 238 and 129, respectively. Each group was divided into normal eyes with AxL > 24.0 mm and eyes with pathologic myopia with AxL > 24.0 mm according to the guidelines defined in IMI. Pathologic myopia is defined as excessive axial elongation associated with myopia that causes structural changes in the posterior segment of the eye (myopic maculopathy, including posterior staphyloma, and high-myopia-associated optic neuropathy) and results in loss of best-corrected visual acuity [20]. In this study, pathologic myopia was considered to be any type of posterior staphyloma and part of categories 2, 3, or 4 of the meta-analysis for the pathologic myopia classification system: diffuse choroidal atrophy (category 2), patchy chorioretinal atrophy (category 3), and macular atrophy (category 4) [10,21]. Three additional indicators were defined, namely lacquer cracks, myopic choroidal neovascularization, and Fuchs spots [22]. Posterior staphyloma was classified according to the definitions provided by Curtin and the IMI [23]. AxL was measured using ocular biometry (IOL Master; Carl Zeiss Meditec, Jena, Germany). The diagnosis of posterior staphyloma and pathologic myopia using stereoscopic fundus photography was determined by two trained ophthalmologists (YCK and CKP). If the results of the two ophthalmologists differed, they made a final decision through discussion. The study protocol was approved by the institutional review board of the Catholic University of Korea (IRB No. OC19RESI0161).

### 2.2. Data Acquisition

**Figure 1:**
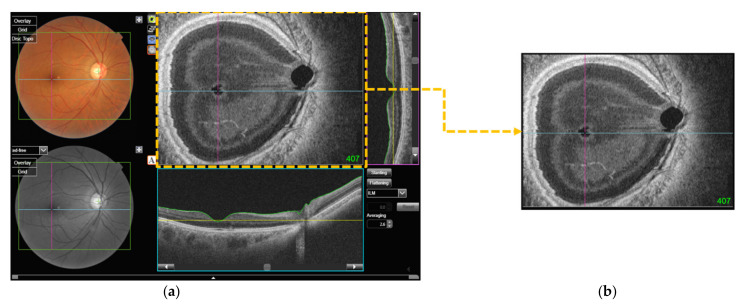

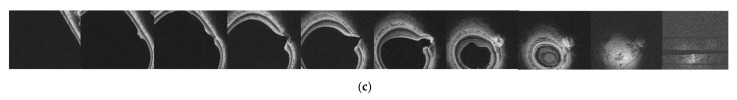
Data acquisition process. (**a**) All OCT C-scans were obtained using OCT Triton. (**b**) The C-scan images are labeled in green at the bottom right, from 1 to approximately 1000. Using the PyautoGUI, we set to pass from the first image to the last image in 5 s, and a video file was created by recording it; (**c**) 150 image frames were extracted from the image, and 10 of them are illustrated.

Image data of the patients were obtained using OCT (DRIOCT Triton; Topcon Corporation, Tokyo, Japan). The collected image is the OCT en face, which creates a front section of the retinal layer and is also known as a C-scan [24]. It consists of approximately 1000 images per eye sliced at 2.6 μm. As the number of images in the OCT en face was different for each eye, the number of OCT images was matched for every eye. Therefore, the image was converted into a video format (.mp4) with a length of 30 frames per second and a duration of 5 s. The video was output as 150 images per eye and the size of the image was 256 × 320 pixels. Figure 1 shows the process of data acquisition.

### 2.3. Model Architecture

**Figure 2:**
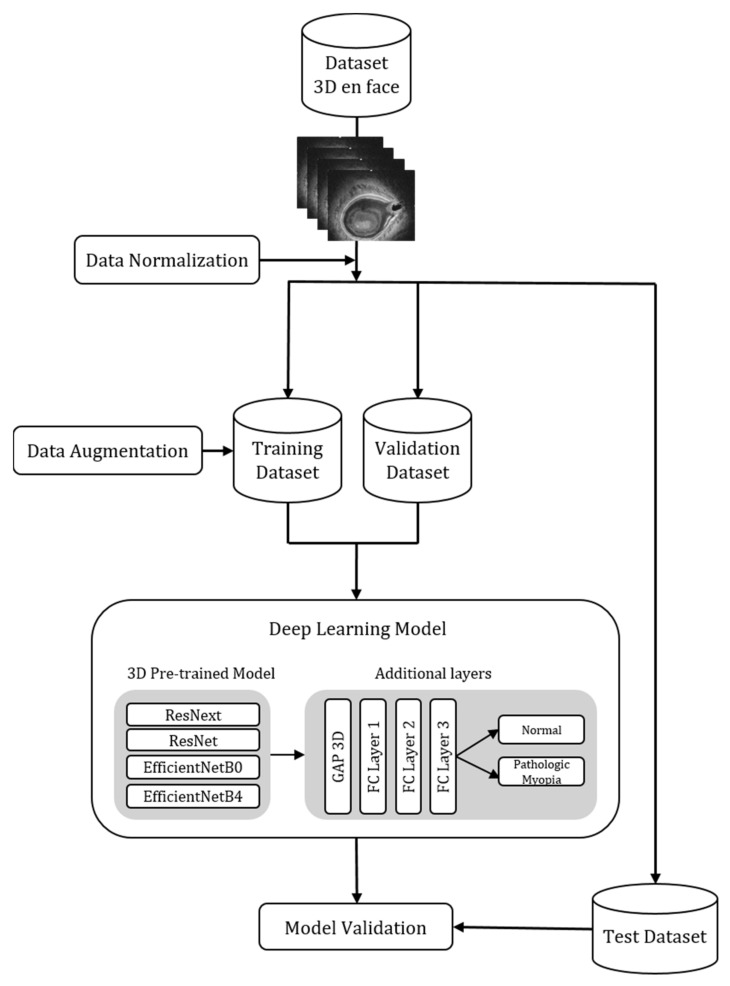
Deep learning model architecture.

Our deep learning model architecture is shown in Figure 2. We used datasets with 367 OCT volumes (150 × 256 × 320 voxels). However, we adjusted the size of the 367 OCT volumes to 100 × 128 × 128 voxels to suit the deep learning model, and the total number of OCT images was 36,700. For the development of deep learning algorithms, the dataset was divided into three groups: training data, validation data, and a test set at a ratio of 8:1:1. All OCT volumes were normalized to have values between zero and one. Since the number of volumes in our training dataset was small, it was increased through data augmentation. We used the RandAugment, among various other data augmentation strategies. This strategy may adjust the regularization strength according to the size of the model and the size of the training dataset, and it shows lower computational complexity than other auto augmentation strategies, such as AutoAugment and FastAutoAugment [25,26]. We used the RandAugment to increase the number of OCT volumes in the training datasets. Augmentation was performed during the process of generating the batch. Data augmentation was performed by randomly rotating the image angle in the training data between −10° and 10°. In addition, horizontal flip, anisotropy, elastic deformation, blur, noise, swap, and gamma methods were used. Data augmentation can artificially increase the size of training datasets to reduce model overfitting [27]. 

In this study, transfer learning was performed by applying a trained 3D CNN model to solve the classification problem. Transfer learning refers to a technology that applies a model learned in a particular domain to a new domain [28]. As transfer learning uses previously trained models, it also has the advantage of being effective when the number of learning data samples is small and the learning speed is fast [29]. 

A pre-trained 3D CNN was used to extract the characteristics of the 3D OCT en-face image. A 3D CNN is a method for learning spatio-temporal features using deep three-dimensional convolutional networks [30]. Therefore, unlike 2D CNNs, which only search for 2D slices, 3D CNNs that can integrate and analyze all information using spatio-temporal information were adopted. The 3D CNN model used in the analysis was developed based on pre-trained ResNet [31], ResNext [32], EfficientNetB0 [33], and EfficientNetB4 [33] models using 1.2 million images from ImageNet. 

Our model aimed to distinguish between the pathologic myopia group and the normal group. That is, the fully connected layer of the pre-trained 3D CNN models was modified to suit our purpose. Global average pooling 3D was applied to the extracted features. Then, the model was built to classify binary classes by adding two fully connected layers with the scaled exponential linear unit [34] activation functions and the last fully connected layer with sigmoid activation functions. We trained the model using the Adam optimizer [35] and the binary cross-entropy cost function. The learning rate was 0.0001 and the batch size was 4. To address the imbalance between classes, when the deep learning model learned data, a method of weighting loss function for each data class was selected. By multiplying the total number of samples divided by the number of classes by the weight, the loss was kept at a similar magnitude. The values of the weights were 0.7710 and 1.4224 in the normal and patient groups, respectively. Assigning weights to each class can contribute to the improved classification performance of the minor class. We used gradient class activation map (Grad-CAM) technology to solve the black box problem of deep learning. Grad-CAM visualizes features extracted during CNN learning through a heat map [36].

### 2.4. Statistical Analysis

The *t*-test and chi-square test were performed on four demographic variables to confirm the difference between the eyes in the pathologic myopia group and the normal group. Statistical significance was evaluated at *p* < 0.05 using R studio (version 1.3). The accuracy, specificity, sensitivity, and area under the receiver operating characteristic curve (AUROC) were calculated to analyze the performance of the model. A test dataset was used to evaluate the model. Tensorflow 2.5.0 [37] was used to train and evaluate the deep learning models and Pytorch 1.9.0 [38] with TorchIO [39] was used for image enhancement, along with the OpenCV [40] package in Python (version 3.8) for image processing. The learning of this model was trained on a GPU Nvidia Tesla P100-PCIE-16GB.

## 3. Results

### 3.1. Demographics

A total of 367 eyes were included in the analysis, including their 3D en face OCT volumetric images and demographic data. We performed a basic statistical analysis to identify the variables that made a difference between the pathologic myopia eye group and the normal eye group. Gender, age, axial length, and symbolic thickness were significantly different between the two groups (Table 1). All 3D volumetric images were randomly divided into training, validation, and test data from 293, 37, and 37 eyes, respectively (Table 2).

### 3.2. Model Performance

For the automatic classification of pathologic myopia, the performance of the model based on four pre-trained models was evaluated using the test dataset (Table 3). Accuracy, sensitivity, specificity, and AUROC were used as evaluation indicators and were calculated as follows:

(1) $$\mathrm{Accuracy}=\frac{TP+TN}{TP+FP+TN+FN}$$

(2) $$\mathrm{Sensitivity}=\frac{TP}{TP+FN}$$

(3) $$\mathrm{Specificity}=\frac{TN}{TN+FP}$$

where TP, FP, FN, and TN represent true positive, false positive, false negative, and true negative, respectively. TP is the number of samples that are actually pathologic myopia. FP is the number of samples that are actually normal but classified as pathologic myopia. FN is the number of the samples that are actually pathologic myopia but classified as normal. TN is the number of the samples that are actually normal.

**Figure 3:**
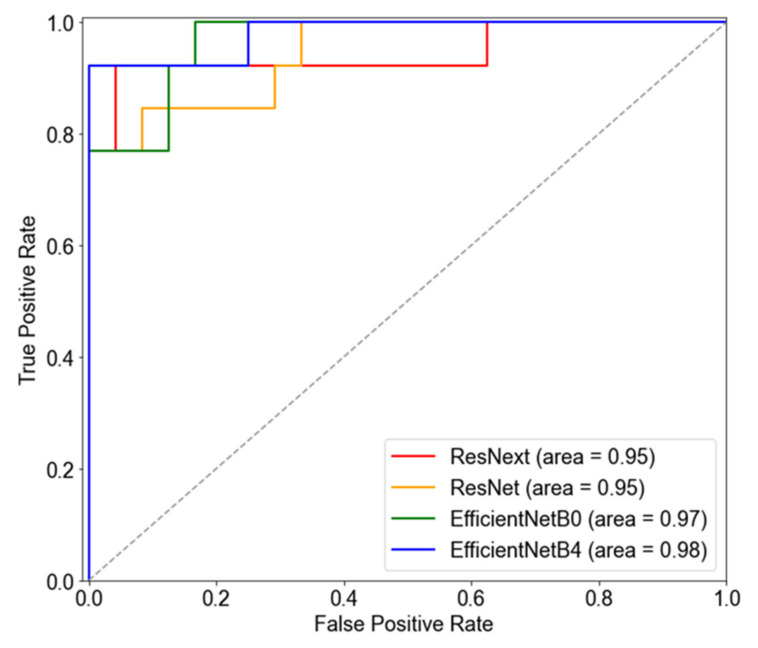
Receiver operating characteristic curves of algorithms for predicting pathologic myopia.

All four models showed an excellent classification performance of 95% or more based on AUROC. In particular, the performance of the EfficientNetB4 based model was the highest, with 95%, 93%, 96%, and 98% for accuracy, sensitivity, specificity, and AUROC, respectively. The ROC curves for the four models are shown in Figure 3. 

**Figure 4:**
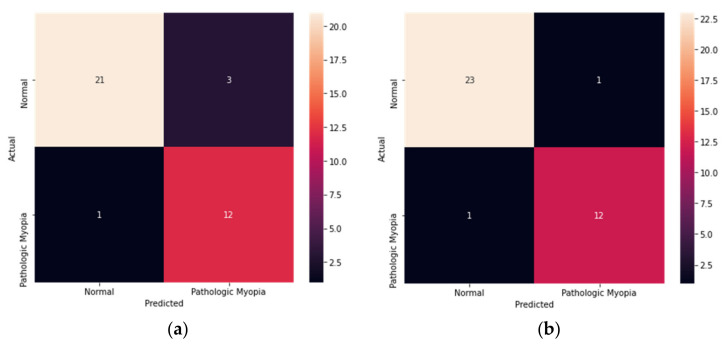
Confusion matrices of the EfficientNetB4 model. (**a**) The confusion matrix of EfficientNetB4 before application of data augmentation. (**b**) The confusion matrix of EfficientNetB4 after application of data augmentation.

The model performances before and after the application of data augmentation were compared. The EfficientNetB4 model, which had the best performance among the four existing models, was also trained without applying data augmentation, and it achieved AUROC of 97% (89% accuracy, 92% sensitivity, and 88% specificity). Figure 4 shows the confusion matrix of the EfficientNetB4 model with data augmentation and the model without data augmentation. After data augmentation, the number of false negatives decreased from three to one. In other words, the application of data augmentation helped improve the model training results.

**Figure 5:**
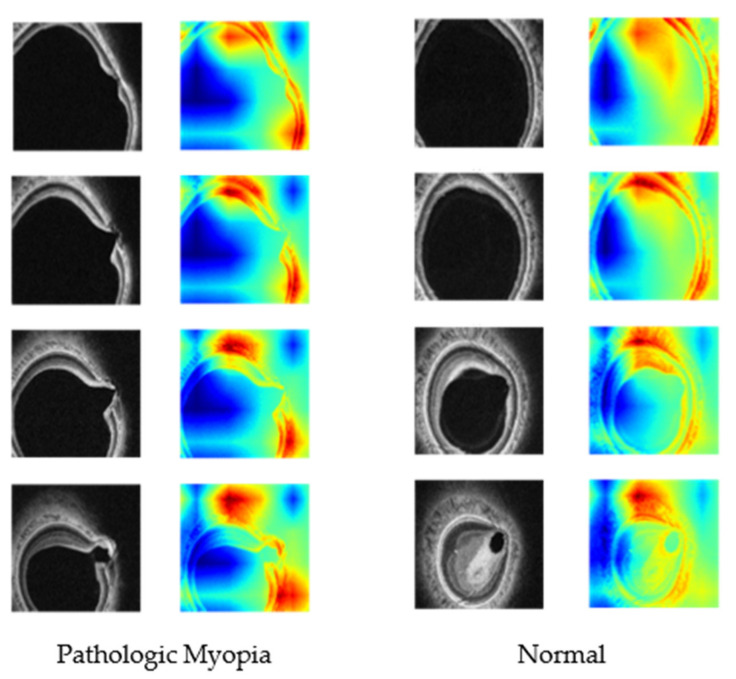
Heat maps using the Grad-CAM techniques for each 3D OCT volume, divided into eyes with pathologic myopia and eyes without pathologic myopia.

Figure 5 shows the results of Grad-CAM for each 3D OCT volume, divided into eyes with pathologic myopia and eyes without pathologic myopia. The results of Grad-CAM showed that differences in the shape (curvature, thickness, location, etc.) of the boundaries which affect the overall shape of the eye, can be associated with the detection of pathologic myopia.

## 4. Discussion

This study is the first to develop a deep learning model that automatically diagnoses pathologic myopia using 3D OCT images. Models that automatically diagnose pathologic myopia based on 3D EfficientNetB4 showed the highest performance with an AUROC 0.98 value, when compared to models based on other 3D CNNs. 

EfficientNet is a deep learning model that focuses on increasing efficiency and accuracy. A high performance may be achieved by appropriately adjusting layer width, layer depth, and input resolution. These three elements are combined at an optimal ratio using the compound scaling method. Thus, it provides a higher accuracy than other pre-trained models [33]. The EfficientNet model ranges from B0 to B7, and it is known that as the number increases from B0 to B7, the number of parameters increases and has a high model accuracy. We applied EfficientNetB4 considering computational speed and computing resources, and it showed a higher performance than other models [41].

The results of Grad-CAM showed that the border area, which affects the shape of the eye, can be characterized in the detection of pathologic myopia. Since we trained the 3D structure of the posterior pole of the eye using the C-scan image without segmentation, the visualization results could serve as a new insight for clinicians. In the future, when clinicians collaborate with AI, they will be able to expand the scope of treatment and contribute to accurate diagnoses.

OCT plays a significant role in the detection of pathologic myopia, by finely detecting structural changes in the posterior globe [42]. However, for an accurate diagnosis, standard photographs are compared based on various detailed examinations and clinical experience. This may not only lead to biased interpretation based on the subjective experience of the clinician, but may also be very time-consuming and costly. The deep learning model developed in this study enables a clearer and easier identification of pathologic myopia because it learns the structure of the posterior globe in 3D without a segmentation process. 

Du R et al. used a deep learning model with 2D fundus images to automatically diagnose pathologic myopia and myopic maculopathy, achieving a 92% accuracy [12]. Our study developed a deep learning model using 3D OCT images and showed a higher performance than previous studies. In addition, OCT has the advantage of extracting information by detecting structural changes in the posterior globe in more detail than the fundus image. 

Kim et al. showed that machine learning algorithms are effective in predicting pathologic myopia [11]. According to this previous study, it was discovered that the relative topographic elevation of the posterior sclera contributes to the classification of pathologic myopia. Therefore, a predictive model was developed using four indicators that manually quantified the relative topography using the three main markers (fovea, optic disc, and deepest point of the eye) of the posterior globe through OCT images. However, the manual measurement of indicators is labor-intensive. Our study showed a higher accuracy, while saving the time and money needed for a diagnosis with a deep learning algorithm, which classifies pathologic myopia using OCT images without segmentation.

Yoo et al. utilized deep learning to predict uncorrected refractive errors using posterior segment OCT images [43]. Our study has some differences from this previous study. Firstly, we did not attempt to predict the refractive powers of individuals; instead, we attempted to predict the pathologic myopia that is correlated but does not have causality. Secondly, our model used different datasets. Yoo et al. used the B-scan OCT data, and we used the C-scan data. Finally, they used each 2D image for the vertical and horizontal axes, while we used a 3D image. In addition, they developed a regression model using 2D CNN, while we developed a classification model using 3D CNN.

Our study has several limitations. Firstly, in this study, only the en face OCT image created in the front section of the retina layer was used. There are some strengths to the deep learning model using C-scan. Most deep learning models using OCT data use B-scan, which gives an original perspective. Kim et al. gave multiple points on the advantages of the C-scan [11]: A C-scan of the eye can locate the deepest point of the eye (DPE), which can also be used as a surrogate to determine optic disc configuration; the DPE location has correlations with glaucoma occurrence and glaucoma survival rate [44]; and a C-scan can give three-dimensional parameters of the posterior segment, serving as a tool for pathologic discovery and quantification [45]. However, recently, an active deep learning model was developed using multimodal data, and its performance is excellent [46]. It is expected that in the future, it will be possible to develop an algorithm that can improve the performance of predicting pathologic myopia diagnosis by analyzing multimodal imaging, such as OCT B-scan and fundus imaging.

Secondly, the size of datasets used for learning was small. Of the 37 eyes in the test set, 13 OCT eyes with pathologic myopia were evaluated for performance. The number of eyes with pathologic myopia was not large, and data from only two hospitals were collected in this study. Predictive performance is expected to improve in subsequent studies when more samples are collected and analyzed. 

Finally, this study was conducted based on patient data from Korea. As pathologic myopia varies in prevalence from race to race, the model may be less accurate when applied to different ethnic groups [47]. Further research with various ethnic data is needed.

## 5. Conclusions

We developed a deep learning model to automatically diagnose patients with pathologic myopia using 3D OCT volumetric images. Among the four pre-trained models, the model based on EfficientNetB4 showed the highest performance. As OCT images are readily available and routinely used, they can be useful as a clinical decision-making system for clinicians to diagnose patients with pathologic myopia. 

## Figures and Tables

**Table 1:** Patient characteristics.

Variables		Normal (*n* = 238)	Pathologic Myopia (*n* = 129)	*p* Value
Sex				<0.001
	Male	153 (64.3%)	57 (44.2%)	
	Female	85 (35.7%)	72 (55.8%)	
Age		51.3 ± 13.3	55.7 ± 15.4	0.006
Axial Length		25.6 ± 0.7	27.7 ± 2.2	<0.001
Choroidal Thickness		259.1 ± 98.3	169.2 ± 98.9	<0.001

**Table 2:** Distribution of patients in the training, validation, and test datasets.

	Total (*n* = 367)	Training Set (*n* = 293)	Validation Set (*n* = 37)	Test Set (*n* = 37)
Normal	238 (64.9)	190 (64.8)	24 (64.9)	24 (64.9)
Pathologic Myopia	129 (35.1)	103 (35.2)	13 (35.1)	13 (35.1)

**Table 3:** Performance metrics of different CNN models.

Model	Accuracy	Sensitivity	Specificity	AUROC
ResNext50	0.89	0.92	0.88	0.95
ResNet18	0.86	0.85	0.88	0.95
EfficientNetB0	0.89	0.92	0.88	0.97
EfficientNetB4	0.95	0.93	0.96	0.98

## Data Availability

The data presented in this study are not publicly available due to privacy restrictions.
